# Protocol for the Yapatjarrathati project: a mixed-method implementation trial of a tiered assessment process for identifying fetal alcohol spectrum disorders in a remote Australian community

**DOI:** 10.1186/s12913-019-4378-5

**Published:** 2019-09-09

**Authors:** Dianne C. Shanley, Erinn Hawkins, Marjad Page, Doug Shelton, Wei Liu, Heidi Webster, Karen M. Moritz, Linda Barry, Jenny Ziviani, Shirley Morrissey, Frances O’Callaghan, Andrew Wood, Mary Katsikitis, Natasha Reid

**Affiliations:** 1 School of Applied Psychology, Griffith University, Gold Coast, Australia; 2 Menzies Health Institute of Queensland, Gold Coast, Australia; 3 North West Hospital and Health Service, Mt Isa, Australia; 4 Women’s and Children’s Health Services, Gold Coast University Hospital, Gold Coast, Australia; 5 Child Development Services, Sunshine Coast Health Services, Sunshine Coast, Australia; 6 Child Health Research Centre and School of Biomedical Sciences, The University of Queensland, Brisbane, Australia; 7 Queensland Statewide Child and Youth Clinical Network, Centre for Children’s Health Research, Brisbane, Australia; 8 School of Health and Rehabilitation, The University of Queensland, Brisbane, Australia; 9 School of Social Sciences, University of the Sunshine Coast, Sunshine Coast, Australia

**Keywords:** Fetal alcohol spectrum disorder, Developmental assessment, Rural and remote areas, First nations, Indigenous communities, Implementation strategy, Aboriginal and/or Torres Strait Islander peoples

## Abstract

**Background:**

Fetal alcohol spectrum disorder (FASD) is a highly prevalent neurodevelopmental disorder associated with prenatal alcohol exposure. Early identification can improve functioning for individuals and reduce costs to society. Gold standard methods of diagnosing FASD rely on specialists to deliver intensive, multidisciplinary assessments. While comprehensive, prevalence rates highlight that this assessment model cannot meet demand, nor is it feasible in remote areas where specialist services are lacking. This project aims to expand the capabilities of remote practitioners in north Queensland, Australia, where 23–94% of the community identify as First Nations people. Integrating cultural protocols with the implementation science theories of Knowledge-To-Action, Experience-Based Co-Design, and RE-AIM, remote practitioners with varying levels of experience will be trained in a co-designed, culturally appropriate, tiered neurodevelopmental assessment process that considers FASD as a potential outcome. This innovative assessment process can be shared between primary and tertiary health care settings, improving access to services for children and families. This project aims to demonstrate that neurodevelopmental assessments can be integrated seamlessly with established community practices and sustained through evidence-based workforce development strategies.

**Methods:**

The Yapatjarrathati project (named by the local First Nations community and meaning ‘to get well’) is a mixed-method implementation trial of a tiered assessment process for identifying FASD within a remote Australian community. In collaboration with the community, we co-designed: (a) a culturally sensitive, tiered, neurodevelopmental assessment process for identifying FASD, and (b) training materials that up-skill remote practitioners with varying levels of expertise. Qualitative interviews for primary, secondary and end users will be undertaken to evaluate the implementation strategies. RE-AIM will be used to evaluate the reach, effectiveness, adoption, implementation and maintenance of the assessment and training process.

**Discussion:**

Co-designed with the local community, integrated with cultural protocols, and based on implementation science theories, the assessment and training process from this project will have the potential to be scaled-up across other remote locations and trialed in urban settings. The Yapatjarrathati project is an important step towards increasing the availability of neurodevelopmental services across Australia and empowering remote practitioners to contribute to the FASD assessment process.

## Background

### Fetal alcohol spectrum disorder (FASD)

FASD is a lifelong neurodevelopmental condition associated with prenatal alcohol exposure and characterised by, but not limited to, physical, emotional, behavioural, cognitive and social impairments [1]. There is an array of secondary conditions that can be associated with FASD (e.g., involvement with the criminal justice system, drug and alcohol dependency, and mental health problems) that result in a significant social and economic burden to individuals, families and society. For example, 36% of youth in Western Australian detention centers met criteria for FASD [2] and a New Zealand study estimated that FASD costs in 2013 ranged from $49 million to $200 million [3]. FASD is quickly becoming recognised as one of the most prevalent neurodevelopmental disorders impacting children, particularly in some remote locations where alcohol consumption is high. Recent FASD prevalence rates in the United States (US) ranged from 1 to 5% using a conservative approach and 3 to 9.9% using a less conservative approach [4].

Despite these alarming rates, FASD continues to be under-recognised and under-diagnosed in Australia [5], partially because practitioners conducting early childhood assessments may not be asking about prenatal alcohol use, but also because gold standard FASD assessments are lengthy, costly, and not easily accessible. Consequently, high rates of missed or misdiagnosis currently exist. Previous research found that 86.5% of a clinical sample of foster and adopted children who met FASD criteria, had not been previously diagnosed with FASD or had been misdiagnosed [6]. Increasing access to efficient, valid assessments would address these missed and misdiagnoses. When children are identified as having a neurodevelopmental disability (e.g., FASD, intellectual disability, autism spectrum disorder), access to disability funding can occur, practitioners can adjust support strategies accordingly, and outcomes can be improved [7]. Early identification and intervention for neurodevelopmental conditions, including FASD can: (a) reduce demands on public services by commencing children on support trajectories earlier in life [8]; (b) provide the opportunity to prevent future pregnancies within a family from being alcohol affected [9]; and (c) result in a significant return for society, including enhanced socio-emotional functioning for individuals, improved school attendance, increased employment, as well as a reduction in child protection and youth justice service involvement [10]. Remote locations in Queensland, Australia do not currently have access to specialised FASD services, and few remote practitioners have been trained in the assessment and support of children with FASD.

### The current assessment process for diagnosing FASD

Specialist assessment services in Australia follow the Australian Guide to the Diagnosis of FASD, which was modelled after US and Canadian assessment guidelines [2]. The comprehensive neurodevelopmental assessments conducted in two urban hospital and health services are considered the ‘gold standard’ assessment model when diagnosing FASD in Queensland because they use a large number of reliable and valid assessment tools selected via expert consensus, and are administered by a multidisciplinary team of specialists. This assessment process, which can require up to 2 days of assessments per child, is followed by a multidisciplinary case-conference to collate assessment outcomes and reach a diagnostic formulation [5]. While this process provides a comprehensive conceptualisation of a child’s abilities, it is labour intensive and costly. The feasibility and cultural sensitivity of this assessment process in remote areas that lack or have limited access to resources and specialist expertise also needs to be considered. Given the prevalence of FASD, and the challenges faced by remote settings, flexible yet valid ways to conduct neurodevelopmental assessments that identify FASD are needed. This study protocol outlines the procedure through which an innovative, culturally sensitive, tiered neurodevelopmental assessment process that considers FASD was co-created with the community and how it will be implemented.

### Development of a tiered assessment process

#### Developing cultural competence

Around one quarter of the community in the principle town identify as First Nations. This town services surrounding communities where up to 94% identified as First Nations. The diversity of nation groups and the low socio-economic status within the region is likely due to the forced removal of people from their lands and the exploitive nature of colonisation on the community. The team recognises the power of colonised privilege and did not want to create another mechanism for subjugation [11]. We aimed to acknowledge the determinants of inequity, and to embed the project within the First Nation worldview and philosophy. To this end, First Nations co-leadership has been part of the project from its inception. As a First Nations rural general practitioner, one of the authors (MP) was instrumental in creating a culturally safe space for the research team to connect with local Elders. Using an ‘all teach, all learn approach [12], Elders travelled to the urban center to meet the research team, and the research team visited the remote community to learn more about First Nations’ worldview. Time was set aside to create space for two-way learning between First Nations and non-First Nations researchers [12]. For example, through yarning or story-telling, and cooking food ‘on country’, local community members and Elders provided cultural competence training to the research team. This included learning about the sacred nature of land and animals, which shaped the assessment process by incorporating local totems. Additionally, the research team recognised that trust, or feeling comfortable, was measured by the quality of relationships and the value of genuine connection, which comes from the heart. Strong ties emerged through weekly and sometimes daily phone conversations between Elders and key research personnel, solidifying the quality of the relationships, despite the distance. Furthermore, the importance of ensuring that First Nations health practitioners support First Nations children and families who participate in the assessment process was highlighted to the research team. Together it was decided the assessment process needed to be holistic, including attention to the physical, spiritual, cultural, emotional and social well-being of the community and fitting in with community capacity.

Specific local cultural protocols will continue to guide the project. This includes recognition of the multiple nation/language groups in the region; skinship and kinship rules relating to customs, processes, roles, and responsibilities of First Nations individuals; knowledge of the person(s) by whom one should be guided about local practices (e.g., community elders); seeking necessary permissions (e.g., permission to conduct the project from the Traditional Owners of the Land); and respecting First Nations lore (e.g., respect for traditional laws referring to common features of acceptable and unacceptable behaviour in First Nations Communities).

Broad cultural protocols outlining how to conduct research with First Nations communities [13–16] have corroborated what has been learned from Elders and will support engagement and implementation. Themes from these protocols include respect for First Nation’s ways and knowledge, co-creating programs with the community, enabling a community governance, incorporating narrative practices (e.g., yarning), focusing on strength-based empowerment, implementing assessments cautiously, and investing time in relationships that demonstrate equality, integrity, respect, responsibility, reciprocity, and cultural continuity. Local and broad cultural protocols provide the overarching guide to ensure the project purposefully seeks and responsively includes First Nation’s knowledge, community characteristics / needs, with evidence-based practice and practical service delivery barriers. Notably, First Nations was the largest cultural group within the remote region; however, the project sought to identify and respect the needs of other local cultural groups including rural, farming and refugee cultures as well as respecting needs that are specific to societal challenges, such as low socio-economic status.

#### The integration of cultural protocols with implementation science

**Fig. 1:**
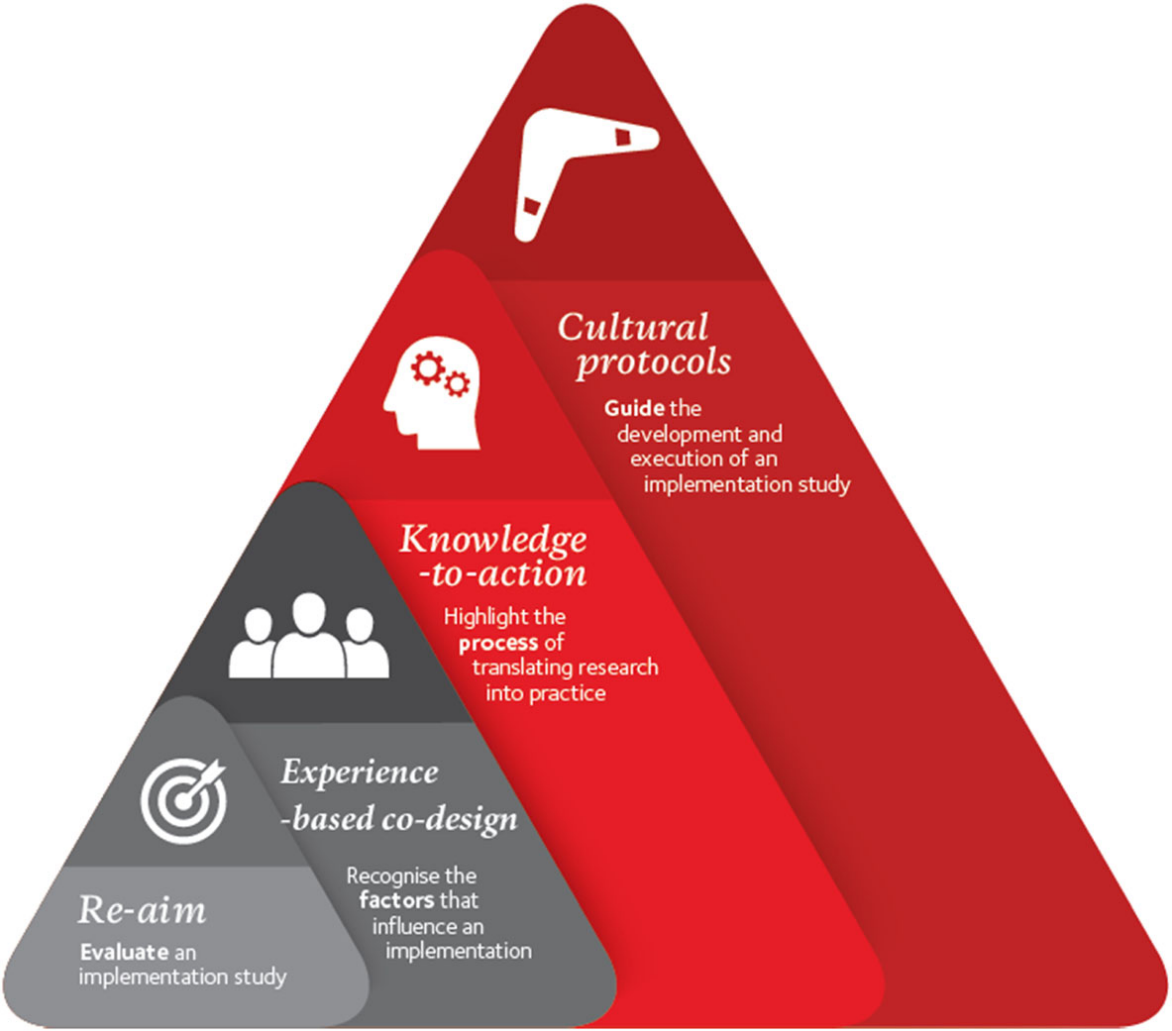
The Integration of Cultural Protocols with Implementation Science Theories

Implementation science is “the scientific study of methods to promote the systematic uptake of research findings and other evidence-based practices into routine practice, and hence, to improve the quality and effectiveness of health services and care”^p.1^ [17]. Due to the recent proliferation of implementation science theories, Nilsen categorised these theories into three specific aims (a): describing the process of transforming research into practice (b); understanding factors that influence the outcomes of an implementation; and (c) evaluating the implementation [18]. The current project adopted Nilsen’s recommendations to identify an implementation theory that would assist with each aim at the outset of the project. The Yapatjarrathati Project adopted: (a) the Knowledge-to-Action (KTA) framework to highlight the *process* of translating research into practice; (b) Experience-based co-design to recognise the *factors that influence* the implementation; and (c) the RE-AIM framework to provide a mechanism for *evaluating* the implementation (See Fig. 1).

*The Knowledge-to-Action (KTA) Framework* outlines two distinct, but related processes for translating knowledge: (a) the knowledge creation phase; and (b) the action cycle [18, 19]. ‘Knowledge Creation’ involves the synthesis of evidence-based information and tools that represent this synthesis (e.g., educational modules, practice guidelines, or decision aids for practitioners). For example, as part of the knowledge creation phase, the project team systematically reviewed international strategies for assessing neurodevelopment broadly, and FASD in particular, to identify how the Australian FASD Guidelines can be flexibly adapted to the limitations inherent in remote communities (i.e., large numbers of children requiring assessment, not enough specialist multidisciplinary teams to provide assessments).

The ‘Action Cycle’ outlines the process of adapting knowledge to the local context and explicitly assessing barriers and facilitators to the use of this knowledge. For example, newly developed educational tools within the assessment process have been co-designed and guided by feedback from the local community. The process of obtaining this feedback has been iterative and non-linear, providing the necessary flexibility to adapt knowledge to the local context.

*Experience-based co-design (EBCD)* is an approach that emphasises collaboration between researchers, service-users, families and staff to improve health services. EBCD incorporates participatory action research, user-centred design, learning theory and narrative-based approaches to change [20]. EBCD follows a general process of: (a) gathering experiences from staff, service-users and family members; (b) identifying ‘touchpoints’ [i.e., crucial moments when a service user (or patient) comes into contact with a service environment (or organisation)]; (c) providing this information to the participants; (d) prioritising the touchpoints; (e) bringing everyone together for a co-design event; and (f) holding a celebration to review what has been achieved [20, 21]. This co-design process places the community’s values and needs at the very heart of the research outcomes. In the current project, one crucial touchpoint was seeking informed consent from caregivers to conduct the child’s assessment. A local, First Nations, rural general practitioner (author MP) developed a dreamtime story that incorporated the research teams’ evidence-based information about neurodevelopmental assessments with culturally appropriate story-telling. This explained the assessment process to families in a culturally appropriate way and is used to seek informed consent from caregivers before the assessment commences. Community members provided feedback on the story and commented that the story helped them better understand FASD as well as the assessment journey.

*RE-AIM* is a widely used framework for evaluating the implementation process [22]. The acronym stands for *Reach*, which provides details on the percentage of people from a given population who participate in a project and describes their characteristics; *Effectiveness*, the positive or negative outcomes of the project; *Adoption*, the percentage of possible organisations and staff that agree to participate in a project; *Implementation*, the process of turning strategies and plans into action, the cost-effectiveness and the fidelity of the project to the original design; and *Maintenance,* which captures the sustainability of the project at both an individual and organisational level [23, 24]. RE-AIM will be used to evaluate the service delivered to the community and the training provided to practitioners to upskill them in this new model of care.

#### Translating co-created knowledge and tools into practice

Empowering remote locations to implement new assessment procedures involves mobilising knowledge that already exists within the community and supporting local practitioners in acquiring new knowledge. One of the most common forms of continuing education for health care workers is workshop attendance. While often cost-effective, attending workshops tends to be relatively unsuccessful at changing practices over the long term [25, 26]. Interactive dissemination strategies (e.g., observing, rehearsing and practicing skills, supervision and peer discussion) are required to create sustained change in usual care and result in better retention of information, practitioner satisfaction, self-efficacy, and use of the techniques learned [27–29]. In the current project, experienced professionals from urban areas already delivering FASD services have offered workshops in the remote location and will deliver one-to-one or small group clinical supervision and mentoring to practitioners by videoconference. This offers the opportunity for staff to engage in reflective practice tailored to their knowledge and to rehearse new skills [30, 31]. When assessments commence, collaborative, inter-professional case-based learning will also be delivered by videoconference across agencies to provide the opportunity to learn from other practitioners across diverse disciplines, develop professional support networks and consolidate new skills. This offers a diverse range of interactive dissemination strategies to maximally support remote practitioners in accessing evidence-based information for complex cases from experts in the field.

## Methods

### Study aims

The overarching aim of the current project is to assess the effectiveness and implementation of an innovative, tiered, culturally sensitive, neurodevelopmental assessment process within remote geographic locations with limited professional expertise. Practitioners, trained and supported through interactive dissemination strategies, will significantly expand the local workforce capacity. This will leave a legacy of sustained change in assessment and support services for children in remote communities with complex neurodevelopmental presentations, including FASD. To date, this project has used KTA theory and EBCD to:

1. Co-create a culturally sensitive, tiered neurodevelopmental assessment process that incorporates consideration of FASD and integrates seamlessly with established community practices in remote Queensland communities.
2. Develop training resources and deliver training to remote practitioners with varying levels of experience, upskilling them in the tiered neurodevelopmental assessment process.

This protocol paper outlines how our team will use the RE-AIM framework to:

1. Implement the tiered assessment process and support local practitioners using evidence-based workforce development strategies, such as providing ongoing tele-mentoring from neurodevelopmental experts.
2. Evaluate the effectiveness and implementation of the Yapatjarrathati Project at every level of the project delivery.

### Study site and population

In 2014, the North Queensland project region had an estimated population of 32,621, with 23.1–94% identifying as First Nations people (compared to 3.1% for Queensland). Almost half the population was classified as being in the lowest category for socioeconomic disadvantage and two specific communities in the region were classified as having more than 85% of the population in the most disadvantaged category. Significant health issues have been documented for people in the project region, which related to poor nutrition, smoking, harmful consumption of alcohol and other drugs, overweight and obesity, physical inactivity, and a range of socioemotional and social well-being factors [32].

### Study design

A mixed-method, pre-post implementation trial [33, 34] was adopted because: (a) there is indirect evidence of effectiveness for tiered assessment processes internationally [35] supporting the applicability to remote settings in Australia, (b) assessment processes currently used in Australian urban settings cannot be translated into remote settings due to financial cost and lack of multi-disciplinary expertise, and (c) a tiered assessment process is considered feasible and acceptable in the targeted remote communities [33].

Adopting three distinct but complementary implementation frameworks within the Yapatjarrathati Project provided the broader benefits of: (a) potentially speeding the translation of research findings into routine care, (b) recognising the complexities and methodological trade-offs necessary when balancing internal and external validity, (c) maximising the clinical utility of research for practicing clinicians, resulting in an end product that lasts longer than the project timeline and a sense of ownership over the product from within the community, and (d) potentially yielding rich data that can have scientific and public health and policy impacts [33].

### Sample size

Primary users of the assessment process (i.e., the target group whose needs are to be prioritised) were defined as specialist practitioners who are trained by the project team (e.g. allied health practitioners, general practitioners, paediatricians). Secondary users of the assessment process (i.e., the group who should generally be satisfied if primary user needs are met, but may require some additional accommodations) were defined as local health workers who have experience working with children and families, but who may not have specialist tertiary education (e.g., health workers, child care workers, child safety officers). End users were defined as the children and families accessing the neurodevelopmental services.

This project will use a census methodology rather than a representative sample for primary and secondary users. We aim to reach all eligible practitioners working in the project region. To be eligible, practitioners will be working in a maternal, child health/mental health or an education setting where training in neurodevelopmental assessment, including FASD could be relevant to their practice. All practitioners (primary and secondary users) in the target regions who meet the inclusion criteria will be invited to participate in the training and complete the pre-post quantitative measures. The minimum number of practitioners required to compare pre- and post- data using a repeated measures ANOVA, with a power of 0.80, an alpha of 0.05 and a medium effect size (0.25) is 28. Primary, secondary and end users will be invited to participate in qualitative interviews. We will consider there to be sufficient data saturation from the interviews when there is repetition in the new data collected, in line with Strass and Corbin [36].

### Procedure

As highlighted, local and general cultural protocols for conducting research with First Nations communities will guide all procedures for engagement, evaluating clinical effectiveness and implementation. Consent to conduct research has been obtained from the regional committee that represents the Traditional Owners of the land. University (GU Ref No: 2018/747) and Hospital Human Research Ethics Committee (HREC/18/QRCH/127) approval has also been obtained. This project did not meet criteria for registration as a clinical trial.

#### The development of a stakeholder group

Project procedures and materials will be discussed at regular intervals with a stakeholder group that has representation from local Elders, First Nations health practitioners, community agencies and practitioners and community members.

#### Qualitative data collection

A convenience sample of primary, secondary, and end users will be interviewed across agencies to evaluate outcomes from the project [interviews will continue until data saturation has occurred (i.e., based on the amount of repetition in the data)]. Yarning will be used as a research method to gather qualitative information with individuals from the stakeholder group, community members, and primary, secondary, and end users. This process of telling stories to share knowledge is recognised as a culturally appropriate method to gather information [37]. Yarning helps facilitate in-depth discussions and provides a culturally safe space for participants to tell their stories. Importantly, through privileging First Nation people’s voices, yarning is a means of changing health outcomes for First Nations Australians [38, 39]. Depending on the stage of the project, qualitative interviews will be conducted to identify: (a) community strengths and needs, (b) current community understanding of FASD, (c) the value of the educational tools, (d) the accessibility of the assessment process (for primary, secondary and end users), and (e) satisfaction with the assessment process. The information yielded will assist with quality enhancements of the assessment process, educational tools, and training resources beyond the research study.

#### Co-creating a culturally sensitive, tiered neurodevelopmental assessment process, that incorporates consideration of FASD

The assessment process has been co-created with the local stakeholder group and project team by combining information synthesised from the FASD literature and current practice guidelines (i.e., knowledge creation), with culturally appropriate materials and qualitative themes that emerged from yarning during the initial engagement phase of the study (i.e., action cycle). The assessment process consists of six tiers. Tier 1 is a culturally sensitive dreamtime story to explain the value of the assessment process to families and to seek informed consent. Tier 2 involves a culturally sensitive developmental interview, a specific measure of alcohol use during the pregnancy (the Alcohol Use Disorders Identification Test-Consumption) [40], and physical measurements [2]. Tier 3 involves the administration of the Rapid Neuro-Developmental Assessment (RNDA) [41], an abbreviated functional assessment that screens for vision and hearing problems and provides information about seven of the ten neurodevelopmental domains required to assess FASD (motor, speech, cognition, attention, hyperactivity and impulse control, adaptive behaviour and social skills, and seizures). Tier 4 involves collecting collateral information from a caregiver and teacher to obtain information about attention, executive functioning, affect regulation, and adaptive functioning. Typically, this involves a structured questionnaire such as the Behavior Assessment System for Children, third edition (BASC-3) [42]. Tier 5 collates information for an initial case formulation and commences the planning of evidence-based intervention strategies with the information already obtained, starting the child on a support trajectory regardless of diagnostic status. This includes a case conference between the family, practitioners, educators and others involved in the child’s care to plan the best support trajectory for the child. Where required, tier 6 ‘drills down’ to provide more in-depth assessment of any of the ten neurodevelopmental domains by specialists, as clinically indicated. This includes additional feedback to key stakeholders as required.

The tiered process provides a soft-entry into the health care system. Community-based professionals (e.g., First Nations health workers, early childhood educators, youth workers, child safety officers) can use Tiers 1–4 as a screener for neurodevelopmental impairment, collecting as much information as possible within the child’s daily environment. This provides the opportunity for health workers and educators to connect in culturally meaningful ways with families and to commence the assessment process inside family homes or schools, and outside of mainstream medical settings. Multiple disciplines contribute to the collation of information (as indicated in the Australian Guide to FASD diagnosis), and a qualified health professional in the primary care system (typically a general practitioner) becomes the central point of care to make a diagnostic decision where possible, or to refer for more comprehensive assessment, as indicated.

#### Training remote practitioners

A tiered training process has been developed to match the assessment process. With the help of the local stakeholder group, initial broad training sessions were provided to practitioners in the community, providing information about the effects of prenatal alcohol exposure on brain development, the benefits of assessment and strategies for support. Service maps of the local area were developed, and community agencies were invited to attend. Training was video recorded and provided as webinars for practitioners who could not attend. Subsequently, detailed training for the tiered assessment process was provided to practitioners who identified at the initial training as interested in participating. Surveys of knowledge, sense of competence and current practices were administered pre and post training sessions and will be re-administered 6 months following training sessions with practitioners.

#### Implementing the tiered assessment process

Community members and/or practitioners will refer children suspected of prenatal alcohol exposure, or children where there are concerns about neurodevelopmental delays, to partner agencies within the community. A local project coordinator will commence the tiered assessment process. This coordinator assists the local general practitioners, who act as the central point of care for collating information from community practitioners, coordinating case conferences, and future referrals to specialists. Any community practitioner who received training in the tiered, culturally sensitive, neurodevelopmental assessment process is eligible for support through a combination of individualised clinical supervision, as well as multidisciplinary tele-mentoring. This support assists primary users in clinical decision making related to neurodevelopmental assessments, and secondary users in understanding their role in the assessment process, the information that is within their scope of competence to collect, and how they can best assist the child, family and GP in providing coordinated local care.

Initially, experts from urban settings already providing FASD assessments will provide regular individualised, tele-mentoring to these local remote professionals. It is anticipated that a local practitioner network will emerge as an outcome of this project, sustainably leaving a competent, supportive network of professional relationships that have developed over the course of supervision and mentoring. This network can continue after the term of funding for the project is completed.

#### Evaluating the implementation trial

RE-AIM will guide the evaluation of the implementation trial. A detailed description of how each RE-AIM domain will be assessed is presented in Table 1.

**Table 1:** Evaluation framework

RE-AIM dimension	Current project definition	Time point measured
Reach	The number, proportion and representativeness of (a) practitioners who participate in each tier of training; (b) practitioners who participate in supervision/mentoring; (c) practitioners who participate in a FASD practitioner network; (d) children who are assessed; (e) caregivers, families and other key stakeholders who receive support, advocacy or follow-up.	All items will be measured post implementation.
Effectiveness	Practitioners: • knowledge regarding (a) risks of alcohol use during pregnancy; and (b) FASD. • practitioner level of confidence and proportion who: (a) routinely ask individuals of reproductive age and/or pregnant women and their support networks about alcohol use; (b) provide individuals of reproductive age and/or pregnant women and their support networks with information about alcohol use; or (c) routinely provide individuals of reproductive age with information about effective contraceptive use if not planning pregnancy • practitioner confidence and proportion who routinely ask parents/caregivers of children presenting with developmental concerns about prenatal alcohol exposure as a part of their developmental assessment • practitioner competence for each tier of the assessment process • participation in assessments or referral for assessments Children and families Qualitative information will be collected regarding: • awareness of FASD • knowledge of child development stages • ability to access neurodevelopmental services • satisfaction regarding the services accessed • changes in child/family functioning reported by caregivers following access to services in the community.	Practitioner items will be measured at multiple stages throughout implementation. Child and family items will be measured post implementation
Adoption	The number, proportion and representativeness of settings that adopted the Yapatjarrathati Assessment Protocol	Measured post implementation
Implementation	The extent to which assessments were delivered according to the prototype, and the extent to which training was delivered as intended; the time and cost involved for both training and assessment. The number of iterations required to finalise each prototype will be documented for (a) educational tools, (b) assessment process, and (c) training process as an indication of fidelity to user-centered design. Qualitative information will be collected regarding the facilitators and barriers to implementation (assessment and training) from multiple perspectives: caregivers and family members; other key stakeholders; government and non-government organisations (NGOs).	Process variables will be measured throughout implementation.
Maintenance	At the setting level: the number, proportion and representativeness of organisations that incorporate tiered assessments into their ongoing usual care. At the individual level: the number, proportion and representativeness of practitioners that (a) continue to implement tiered assessments; (b) provide referrals for neurodevelopmental assessments training; engagement in practitioner network 6-months post-training At the community level: Awareness and ability to access Yapatjarrathati Project services	All items will be measured at 6 month follow up.

### Project timeframe

**Fig. 2:**
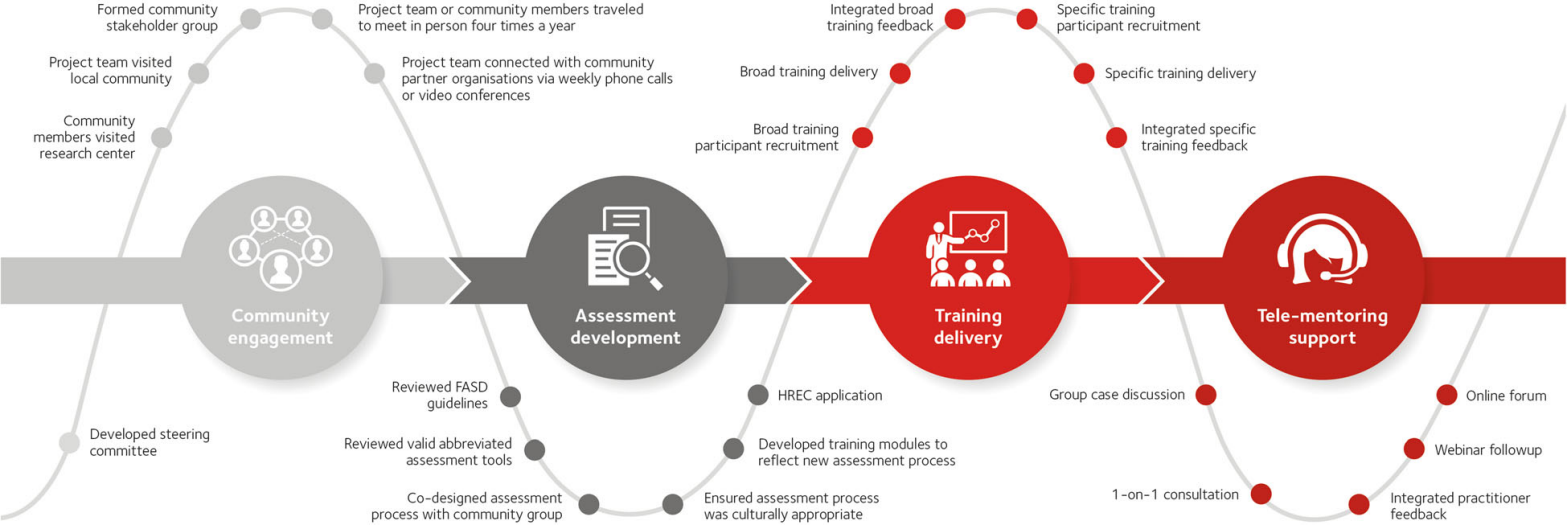
The Journey of the Yapatjarrathati Project

The project began in 2018 and will continue to 2020. The first 6 months represented the engagement phase during which relationships with the community were developed following an ‘all teach, all learn’ philosophy [12] and the local stakeholders’ group was established. Following the engagement phase, the assessment process was co-designed with the community. Specific training in the culturally sensitive, tiered neurodevelopmental assessment process began in 2019. Follow-up tele-mentoring of those implementing the assessment process will continue for the duration of the project. The projected journey for this project is summarised in Fig. 2.

## Discussion

Only 10% of studies provide an explicit rationale (or theoretical framework) for how and why an implementation study will impact proposed outcomes [43]. This article documents the frameworks that have guided the Yapatjarrathati Project, providing a blueprint for future studies interested in linking cultural protocols with implementation science theories to co-create new models of care. To create an effective collaboration with the First Nations community, it is vital that our project fits within a First Nations’ world view; that co-leadership, partnership, engagement, and consent are an integral part of the project; and that broad cultural protocols for conducting research with First Nations communities, as well as specific local protocols guide the development, implementation, data collection, data analysis and dissemination of project results. This is the first project to provide a strategy for integrating cultural protocols with three distinct implementation science frameworks that target different aspects of the implementation process (i.e., knowledge-to-action, experience-based co-design, and RE-AIM). To offer maximal benefit to the community, an implementation trial offers the most efficient process of translating research findings into routine practice while still evaluating effectiveness [34, 35].

This project highlights how we can work together with local communities to embrace concepts of First Nations wellbeing, knowledge and sovereignty; use a strengths-based approach; and build a healthy future together. By listening to and learning from the community, our research team has been challenged to consider: (a) what will be the best process for neurodevelopmental assessments that consider FASD in remote communities; (b) what is necessary and sufficient to commence the trajectory of support for children who are suspected of having neurodevelopmental problems; and (c) how we can enable primary health care to play a significant role in the assessment process to reduce burden on specialist care and increase access to support for this highly prevalent, under-serviced problem.

At the conclusion of this three-year project, the outcomes from the evaluation will provide evidence for a tiered, culturally appropriate neurodevelopmental assessment and training process that can be implemented by practitioners outside of a specialist multidisciplinary setting. Once evaluated, the standardised protocol will provide a robust and portable model of care that can be scaled-up across new settings. As current Australian FASD assessments are undertaken in specialist multidisciplinary settings, this will be the first project to effectively shift assessments out of the tertiary care system into the primary care system within Australia, empowering remote practitioners to provide FASD-related services, and reducing the need for fly-in, fly-out specialists. Given that FASD is under-recognised and consequently under-diagnosed in Australia, the outcomes of this project will be an important step towards increasing the availability of early detection and early intervention for FASD in Australia.

## Data Availability

Data sharing is not applicable to this article as no datasets have been generated yet.

## Abbreviations

BASC-3: Behavior assessment system for children, third edition
EBCD: Experience-based co-design
FASD: Fetal alcohol spectrum disorder
KTA: Knowledge-to-action
RE-AIM: Reach, effectiveness, adoption, implementation, maintenance
RNDA: Rapid neuro-developmental assessment
